# Ferroelectric Domain Structure and Local Piezoelectric Properties of Lead-Free (Ka_0.5_Na_0.5_)NbO_3_ and BiFeO_3_-Based Piezoelectric Ceramics

**DOI:** 10.3390/ma10010047

**Published:** 2017-01-07

**Authors:** Denis Alikin, Anton Turygin, Andrei Kholkin, Vladimir Shur

**Affiliations:** 1 School of Natural Sciences and Mathematics, Ural Federal University, Ekaterinburg 620000, Russia; denis.alikin@urfu.ru (D.A.); anton.turygin@urfu.ru (A.T.); kholkin@ua.pt (A.K.); 2 Department of Physics, CICECO-Aveiro Institute of Materials, University of Aveiro, Aveiro 3810-193, Portugal

**Keywords:** KNN, BFO, MPB, PPB, domain structure, piezoelectric properties, local switching

## Abstract

Recent advances in the development of novel methods for the local characterization of ferroelectric domains open up new opportunities not only to image, but also to control and to create desired domain configurations (domain engineering). The morphotropic and polymorphic phase boundaries that are frequently used to increase the electromechanical and dielectric performance of ferroelectric ceramics have a tremendous effect on the domain structure, which can serve as a signature of complex polarization states and link local and macroscopic piezoelectric and dielectric responses. This is especially important for the study of lead-free ferroelectric ceramics, which is currently replacing traditional lead-containing materials, and great efforts are devoted to increasing their performance to match that of lead zirconate titanate (PZT). In this work, we provide a short overview of the recent progress in the imaging of domain structure in two major families of ceramic lead-free systems based on BiFeO_3_ (BFO) and (Ka_0.5_Na_0.5_)NbO_3_ (KNN). This can be used as a guideline for the understanding of domain processes in lead-free piezoelectric ceramics and provide further insight into the mechanisms of structure–property relationship in these technologically important material families.

## 1. Introduction

Piezoelectric materials exhibit a unique ability to expand under external electric field or to develop a charge under applied mechanical stress, combining high coupling coefficients with exceptional stability and low cost. They are used in a variety of devices, including resonators, ultrasound generators, and actuators realizing precise nano-motion [1,2,3,4,5]. The most widely used materials are currently lead zirconate titanate (PZT)-based ceramics, which are well-known for their excellent piezoelectric properties. However, considering the toxicity of lead and its derivatives, there is a general trend for the development of environmentally-friendly lead-free materials as regulated by several incentives of the European Union. The international efforts in removing toxic substances from everyday applications have been pursued in the last decade. The EU passed the “Waste Electrical and Electronic Equipment” (WEEE) and “Restriction of the use of certain Hazardous Substances in electrical and electronic equipment” (RoHS) initiatives in 2003 [6]. While the WEEE regulates the disposal, reuse, and recycling of the aforementioned devices, RoHS requires that this can be accomplished safely without endangering the environment or people’s health.

As a result, two classes of piezoelectric materials are now being considered as potentially attractive alternatives to PZTs for specific applications: (i) perovskites—i.e., (Bi_0.5_Na_0.5_)TiO_3_ (BNT), BaTiO_3_ (BT), BiFeO_3_ (BFO), KNbO_3_, NaNbO_3_, and solid-solutions on their base; (ii) non-perovskites—i.e., bismuth layer structured ferroelectrics and tungsten-bronze-type compounds. However, their piezoelectric properties were found to be inferior compared to those of PZT. It was shown that—similarly to PZT—the properties could be improved by forming solid solutions of KNbO_3_ with NaNbO_3_ (KNN), so as to sinter ceramics with a composition close to the region of structural instability known as the polymorphic phase boundary (PPB) regulated by the temperature and the morphotropic phase boundary (MPB) controlled by doping. Similar to PZT, the properties of ceramics are also governed by the domain structure and the grain morphology, which are in turn determined by the defect transport and controlled by the sintering method. Such complex interplay of the physical and chemical properties of lead-free piezoelectric ceramics has been recently rationalized in terms of domain wall conductivity and the diffusion of charged defects [7]. Taking recent progress in domain visualization [8,9] and structure refining into account, we believe that the review on the domain-related properties of lead-free piezoelectric ceramics such as BFO and KNN solid solutions is quite timely and can be useful for the specialists working in this field. We will start with a short description of the sintering methods and phase diagrams of these ceramics, and continue with the review of the modern domain visualization techniques. In the following, we will overview the current status of domain studies in BFO and KNN ceramics, and will end with an analysis of the effect of domain structure on the physical properties of ceramics, such as switching behavior and dielectric constant.

## 2. Sintering and Crystal Structure

### 2.1. BiFeO_3_ System

**Figure 1:**
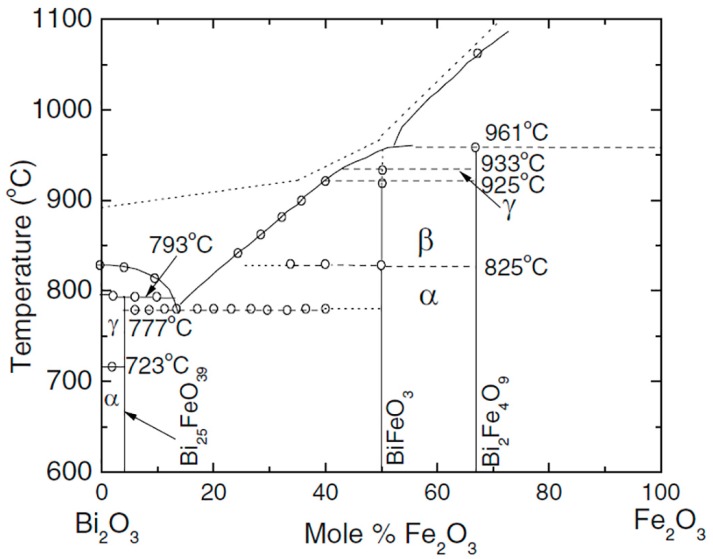
Phase diagram of the Bi_2_O_3_–Fe_2_O_3_ system. Open circles show the data points obtained by differential thermal analysis (DTA). The dotted line above the liquidus represents the approximate temperature limit not to be surpassed in order to avoid decomposition, otherwise correct equilibrium DTA peaks are no longer observed upon a second heating. The α, β, and γ phases are rhombohedral, orthorhombic, and cubic, respectively. Adapted from [23], with permission from © 2008 American Physical Society.

Bismuth ferrite oxide (BFO) is a well-known room temperature multiferroic material belonging to the space group *R3c* [10]. It is attractive due to its very high value of spontaneous polarization of about 100 μC/cm^2^ [10]. However, BFO prepared by a solid state synthesis is typically characterized by a high leakage current due to the high concentration of Fe^2+^ ions and oxygen vacancies [11]. Therefore, it is difficult to polarize the ceramics in order to obtain sufficiently high piezoelectric and dielectric properties. The phase diagram Bi_2_O_3_–Fe_2_O_3_ shows the existence of two equilibrium phases (Fe and Bi saturated): orthorhombic Bi_2_Fe_4_O_9_ (*Pbam*), rhombohedral perovskite BiFeO_3_ (*R3c*), which decomposes to Bi_2_Fe_4_O_9_ and liquid phase at 935 °C, and cubic Bi_25_FeO_39_, which decomposes to Bi_2_O_3_ and liquid phase at 790 °C [12] (Figure 1). A number of experimental and theoretical works have shown the temperature instability of BFO at T > 700 °C with decomposition to Bi_2_O_3_, Fe_2_O_3_, and Bi_2_Fe_4_O_9_ [13]. It has been shown that in the temperature range from 447–767 °C, Bi_25_FeO_39_ and Bi_2_Fe_4_O_9_ phases are more thermodynamically stable than BiFeO_3_, and three phases can coexist [11]. During ceramics synthesis in the temperature range 447–767 °C, BiFeO_3_ partially converts into other phases [14]. The reaction is reversible, and additional annealing returns a BiFeO_3_ phase. A multi-phase state is detected experimentally in a much wider temperature range that can be explained by the small change of Gibbs potential being the driving force for the reaction [15]. Moreover, it has been shown by Valant et al. [16] that the multi-phase ceramic state may be a result of interaction with different types of impurity oxides (AO*_x_*). Different doping strategies (alkaline earth ions, rare earth ions, and others) have been used for the formation of MPBs resulting in the reduction of leakage currents, and therefore, improvement of the ferroelectric and piezoelectric properties [17,18,19,20,21,22].

### 2.2. KNbO_3_–NaNbO_3_ System

**Figure 2:**
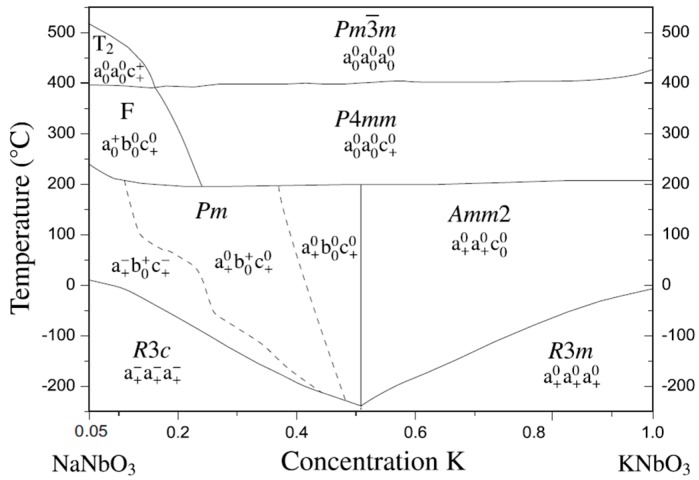
Phase diagram for the system KNbO_3_–NaNbO_3_. Adapted from [32], with permission from © 2009 AIP Publishing LLC.

(K, Na)NbO_3_ is a solid solution of ferroelectric KNbO_3_ and antiferroelectric NaNbO_3_, exhibiting sufficiently high Curie temperature (T_c_ = 420 °C), good ferroelectric properties (P_r_ = 33 μC/cm^2^), and large electromechanical coupling factors [24]. The phase diagram for the KNN system is shown in Figure 2. At room temperature, MPBs lie at 17.5%, 32.5%, and 47.5% NaNbO_3_ contents. It is notable that phase transition temperatures between ferroelectric phases at ~200 °C and between ferroelectric and paraelectric phases at ~400 °C are almost independent of the composition (in contrast to the composition-dependent transition temperature of PZT). Only small substitutions of sodium for potassium in NaNbO_3_ cause a transition to ferroelectric from pure antiferroelectric sodium niobate [25,26]. The piezoelectric data for the air-fired samples are around d_33_ = 80 pC/N, and density of the sample is around 4.25 g/cm^3^ [24,27]. One of the main obstacles for the development of potassium sodium niobate solid solution (KNN) as a commercial piezoelectric ceramic material by conventional method is the difficulty in processing and densification. Egerton and co-workers reported the electrical properties of KNN, in which they indicated relatively low dielectric constants over a wide compositional range [24]. Hence, to achieve sufficient densification, hot-pressed KNN ceramics (~99% of the theoretical density) have been reported to possess a high Curie temperature (T_c_ = 420 °C), a large piezoelectric longitudinal response (d_33_ = 160 pC/N), and a high planar coupling coefficient (k_p_ = 45%). KNN samples have been prepared by conventional air sintering in order to reach high densities (over 95%), which yielded superior piezoelectric properties (d_33_ = 100 pC/N) as compared to those obtained by the same method [28]. It is important to note that KNN material prepared by spark plasma sintering showed significantly higher dielectric and piezoelectric properties than those prepared by conventional method (ε ~ 700 and d_33_ ~ 148 pC/N) [29,30]. Saito et al. fabricated textured-based KNN ceramics by the reactive grain-growth method, which resulted in d_33_ value as high as ~416 pC/N [31].

Furthermore, the volatility of potassium oxide makes it difficult to maintain stoichiometry and high density of ceramics [33]. In order to optimize the processing conditions and to obtain reproducible properties, KNN ceramics are doped with suitable elements: Li^+^ and alkali-earth ions, especially Sr^2+^. The doping changes the cell parameters, promotes densification, decreases the phase transition temperatures, and improves the electrical properties [34,35,36,37]. The addition of LiSbO_3_ or LiTaO_3_ to KNN leads to sufficient enhancement of the dielectric, piezoelectric, and ferroelectric properties of ceramics [38,39]. For more details, we refer the reader to the following exhaustive reviews [33,40,41].

## 3. Methods of Domain Structure Visualization

Rapid development of microscopy techniques brought a lot of possibilities for domain observation in ferroelectric materials [8]. The domains can be visualized by several methods, including optical microscopy, scanning electron microscopy (SEM) with electron backscatter diffraction (EBSD) [42], Transmission electron microscopy (TEM) [43], X-ray diffraction [44,45], and various scanning probe microscopy (SPM) techniques [46,47,48,49], such as piezoresponse force microscopy (PFM) [50], confocal Raman microscopy (CRM) [51], and electric force microscopy (EFM) [48]. The most useful methods for domain visualization in ceramics with high spatial resolution are SEM after selective chemical etching, TEM, PFM, and CRM.

### 3.1. Scanning Electron Microscopy after Selective Chemical Etching

**Figure 3:**
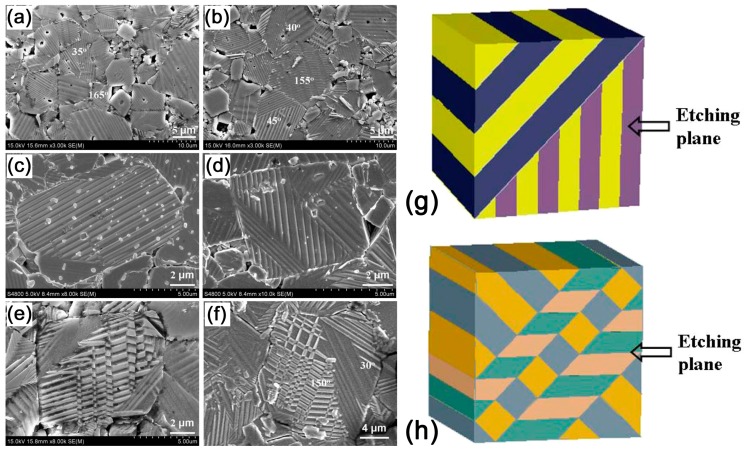
SEM images of domain patterns for the poled NaNbO_3_ (KNN) ceramics. The magnifications are (**a**,**b**) ×3000; (**c**,**e**) ×8000; (**d**) ×10,000 respectively; (**f**) The partially enlarged view of SEM image. Models of domain configurations with the front planes as the observation planes: (**g**) Model for domain configuration type I; and (**h**) for domain configuration type II. The grains with domain configuration type I are in (**c**,**d**), grains with domain configuration type II are in (**e**,**f**). Adapted from [67], with permission from © 2013 AIP Publishing LLC.

Surface chemical etching was the earliest method for the visualization of the static domain structure in ferroelectric single crystals and ceramics [52,53]. This surface treatment is based on the different etching rates of the opposite polarities of a polarization dipole [52,54]. Typically, several acids (HF, HCl, HNO_3_) and alkalis (NaOH, KOH) are used as etchant solutions, depending on the material [55,56,57]. Domains were visualized in BFO after etching with HNO_3_ at room temperature for 2–4 h [55] or with 0.5% HF at room temperature for 45 s [58]. KNN ceramics were chemically etched in 48% HF solution at room temperature for five minutes [59]. The resulting nm-scale change of the surface relief can be visualized with different microscopy methods [60,61,62]. The spatial resolution of the etching technique depends on the visualization method and is typically below 2 nm in SEM registration scattering of electrons from the surface relief (Figure 3a–f). The disadvantages of this method are its destructive influence on the sample surface and possible partial back-switching of domain structure upon etching [63]. However, it is frequently used together with EBSD to get information on domain orientations in randomly-oriented ceramic grains [64,65,66].

### 3.2. Transmission Electron Microscopy

TEM has a very high spatial resolution (below 1 nm), but requires very thin samples; thus, it is very sensitive to the sample preparation [68]. Domains can be irreversibly modified upon polishing and focused ion beam etching. TEM can be used for the imaging of domains, domain walls, and local phase distribution in different piezoelectric ceramics, including BFO and KNN [69,70]. Contrast in this method is provided by different mechanisms, such as the scattering of high energy electrons in the local electric and stress fields [71]. Scanning transmission electron microscopy (STEM) in aberration corrected mode coupled with electron-energy loss spectroscopy can be used for the direct measurement of the atomic displacement in domain wall regions and investigation of defect structure and local strains [7,72,73]. It is of particular importance for ceramics, because the relation between defects (such as dislocations) and domain configurations can be easily identified. Defects and disorder at the grain boundaries can be also seen by TEM. Modern TEM microscopes provide the possibility of directly applying an electric field in the microscope camera, and, therefore, to study domain kinetics in situ [74].

### 3.3. Piezoresponse Force Microscopy

PFM is one of the most useful methods for the visualization of the domain structure in ferroelectric materials, due to its simple sample preparation, generally no need of vacuum or other special conditions, high signal-to-noise ratio, nanometer spatial resolution, and variety of different spectroscopic modes allowing the measurement of local material ferroelectric and dielectric properties [50,75]. PFM is a strain-based scanning probe microscopy [76,77] mode, where application of a modulated electric field to the conductive SPM tip results in the appearance of in-phase surface displacement in a pm–nm range. A lock-in detection technique in different variations is used for the measurements of amplitude and phase of piezoresponse [78]. The phase signal can be linked to the spontaneous polarization orientation, while PFM amplitude is a function of local effective piezoelectric coefficient [79]. PFM allows not only the visualization of domain structure, but also the quantitative determination of the polarization orientation by simultaneous analysis of out-of-plane and in-plane piezoresponse signals (Vector PFM [80], 3D-PFM [80], and angle-resolved PFM [81]). Application of a high enough DC bias to the SPM tip (higher than the threshold field for the polarization reversal) can reverse the spontaneous polarization direction locally in the area under the tip [82]. This allows the measurement of local hysteresis loops of material and the study of domain wall motion in the electric field of the probe. These measurements were quite rarely done on ceramics [77] because of the unknown orientation of a particular grain and interception with the grain boundaries. However, it was possible to determine the intragrain domain wall velocity and other parameters, such as dimensionality of domain walls [83].

### 3.4. Confocal Raman Microscopy

**Figure 4:**
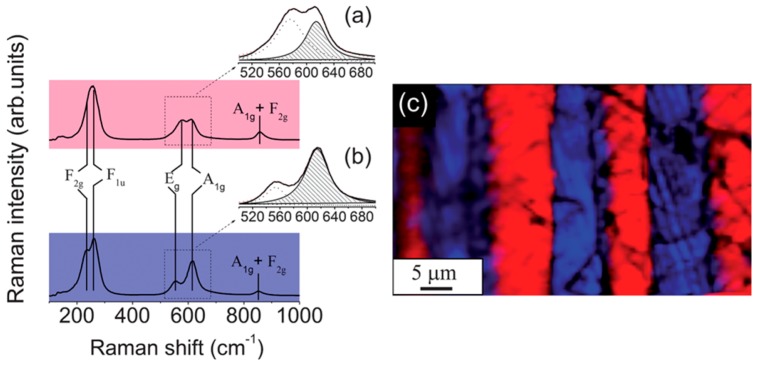
Characterization of KNN ceramics by confocal Raman spectroscopy: (**a**,**b**) Average Raman spectra of adjacent striped domains separated by a 90° domain wall. The insets show magnified Raman spectra and Lorentzian fits of domain structure in the frequency range between 500 and 700 cm^−1^. These spectra are fitted to the sum of two Lorentzian peaks, ascribed to the E_g_ (υ_2_) and A_1g_ (υ_1_) Raman modes, respectively; (**c**) Raman map of domain structure of the KNN exhibiting clear differences between average spectra of adjacent striped domains separated by a 90° domain wall. The Raman map was derived by summing up the total spectral pixel intensity from 100 to 1000 cm^−1^. Adapted from [59], with permission from © 2012 Royal Society of Chemistry.

CRM is based on the study of the Raman spectra variations across the material surface, with spatial resolution of about 300 nm provided by confocal microscopy [84]. The significant change of the Raman spectra (shifts and change of the intensity of the Raman bands) in the vicinity of the domain walls was shown in single crystals, thin films, and ceramics [9,51]. Moreover, the method allows not only the imaging of domain structure (Figure 4) [51], but also the extraction of information about mechanical stresses [85,86] and defect concentration [51]. Polarized Raman scattering can yield knowledge about the orientation of spontaneous polarization in distinct grains of ceramics [9,87].

## 4. Domain Structure in BiFeO_3_

### 4.1. Undoped BiFeO_3_

**Figure 5:**
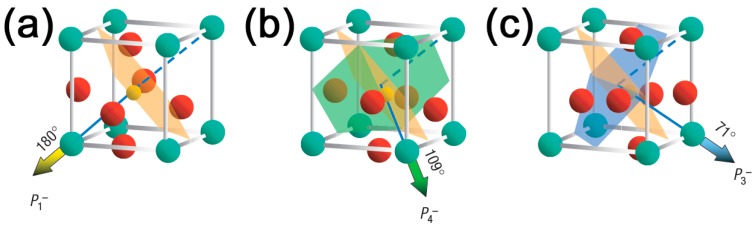
The schematic diagram of (001)-oriented BiFeO_3_ crystal structure and direction of the spontaneous polarization corresponding to the (**a**) 180°; (**b**) 109° and (**c**) 71° domains. Adapted from [88], with permission from © 2006 Nature Publishing Group.

The spontaneous polarization in BFO is oriented along the equivalent crystallographic direction {111} and has eight possible orientations. Based on this, three types of domain walls are possible in BFO: 180°, 109°, and 71° (Figure 5). These angles are the rotation angles between neighboring domains. The permissible domain wall orientations are, therefore, {110} for 109°, {001} for 71°, and any plane parallel to the polarization vector for 180° domains. In a non-perfect crystal, however, it can be expected that the actual wall may deviate slightly from the crystallographically-predicted planes. The smaller the wall area, the larger the possible deviation. Four domain boundary types or configurations are possible, where “head-to-head” or “tail-to-tail” domain walls are charged, while “head-to-tail” or “tail-to-head” walls are non-charged (can be considered neutral).

**Figure 6:**
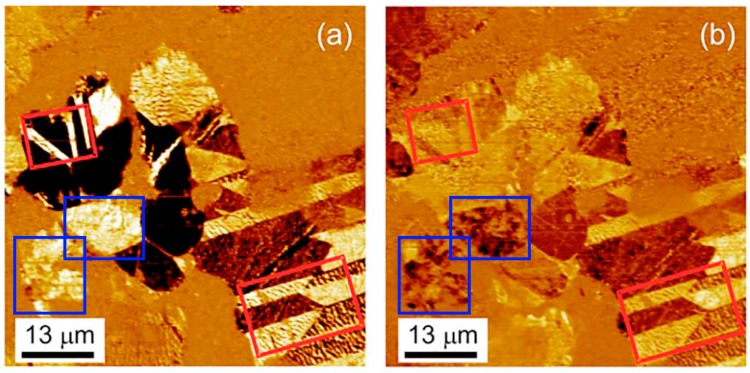
(**a**) Out-of-plane and (**b**) in-plane PFM images of lamellar (red squares) and blotch (blue squares) domain structure in BiFeO_3_. Adapted from [89], with permission from © 2007 AIP Publishing LLC.

Domain structure in BFO ceramics typically represents a mixture of domains with irregular shapes separated by 180° walls (so called, watermarks) and regular lamellar domains separated by non-180° walls (Figure 6) [89]. Being up to 10 μm wide, regular domains are an order of magnitude larger than those reported for BFO thin films [89]. PFM contrast corresponding to the domain structure varies significantly among the grains, as expected for non-oriented ceramics. The nanoscale domain structure at the boundary intersections observed in BFO by TEM can lead to high mechanical stress [90]. Recently, the existence of meta-stable polarization states and vortex structures has been shown in BFO ceramics produced by mechanochemical activation [91]. The domain sizes are typically much smaller than the grain sizes, and domain contrast of as-grown ceramics reflects the intricate interplay of mechanical stresses, uncompensated charges, and defects accumulated at the grain boundaries [92].

### 4.2. Doping by Rare Earth Ions

Commonly, the influence of rare earth element doping on the domain and phase structure results in: (1) transformation of the periodical domain structure to speckle-like domains with smaller sizes; (2) appearance of non-polar/antipolar phase clusters (PFM signal is close to noise with increasing doping level.

**Figure 7:**
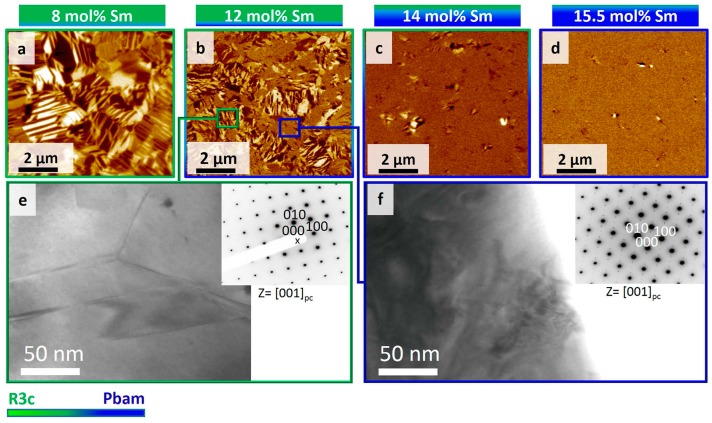
Dependence of the domain structure on Sm^2+^ doping fraction. (**a**–**d**) PFM out-of-plane piezoresponse images obtained for 8 mol % Sm, 12 mol % Sm, 14 mol % Sm, and 15.5 mol % Sm, respectively; (**e**,**f**) Bright field (BF)-TEM images and associated selected area electron diffraction (SAED) patterns from 12 mol % Sm. (**e**) A region identified as *R3c* by corresponding SAED in [001] pc zone axis (inset), where regular domains are seen. (**f**) A region identified as *Pbam* phase by corresponding SAED (inset), where complicated nano-sized features are observed. The green-to-blue color transition approximately represents the change in wt % ratio of *R3c* and *Pbam* phases. PC denotes pseudocubic notation [92].

TEM and PFM studies of Bi*_x_*Re_1-*x*_FeO_3_ (Re = Sm, Gd, Dy) demonstrated concerted change of both crystal structure and piezoresponse contrast distribution [21]. The change of the ferroelectric domain structure and phase composition as a function of the doping level was demonstrated for Sm-doped BFO (8%–18%) [92]. At 8 mol % Sm, the domain structure is mainly comprised of regular lamella and wedges of about 100–500 nm in size, similar to those observed in pristine BFO. As the Sm content is increased to 12 and 14 mol %, the domains become progressively smaller (~50–200 nm) and more irregularly shaped. The number of visible domains is also reduced as the composition approaches the MPB (ranging from 8 to 14 mol % Sm), to the extent that almost no domains are visible at 15.5 mol % Sm (Figure 7). However, the regions with piezoelectric contrast keep a switchable behavior typical of ferroelectric materials. At the same time, the phase concentration significantly depends on the ceramic preparation method [93]. PFM contrast was attributed to consecutive *R3c–Pbam–Pnma* phase transformations. A similar effect of doping was revealed by TEM in Nd- and Sm-doped BFO compositions [94,95]. The compositions with x ≤ 10% were rhombohedral with *R3c* symmetry, and exhibited superstructure and orientational and translational domains characteristic of an antiphase-tilted ferroelectric perovskite [95]. At the phase boundary between the orthoferrite and rhombohedral cells in Nd- and Sm-doped systems, a new structure is stabilized with a quadrupled unit cell similar to the case of PbZrO_3_ [95].

Local piezoresponse in Dy-substituted BFO is approximately three times weaker than in undoped ceramics, thus pointing to a smaller value of the spontaneous polarization and effective piezocoefficient [96]. This can be attributed to the large difference in ionic radii of Bi^3+^ and Dy^3+^ ions, hampering the formation of homogeneous solid solutions. The coexistence of the regions demonstrating a distinct PFM contrast with the areas showing a zero piezoresponse was observed for the x = 0.15 compound.

The piezoresponse in Bi_0.9_Gd_0.1_FeO_3_ was approximately two times weaker as compared to undoped BFO ceramics, due to a smaller value of the spontaneous polarization [97].

### 4.3. Doping by Alkaline Earth and Heavy Metal Ions

Domain structure of BFO ceramics with heterovalent substitution by Ca, Sr, Pb, and Ba ions was studied in Reference [17]. The domain structure represented mostly non-oriented speckle-like domains. At the same time, the average domain size strongly depended on the sintering conditions [89].

Mn-doped BFO samples exhibit a higher volume density of the domain walls than those of undoped ones, suggesting that the Mn ion can effectively reduce the domain size in BFO [98]. 

Negative self-polarization (or polarization offset) was found in the Pr and Sc co-substituted Bi_0.9_Pr_0.1_Fe_1−*x*_Sc*_x_*O_3_ (0.01 ≤ *x* ≤ 0.07), which had a maximum for about 3% Sc and 2% Pr [99]. Negative self-polarization in these samples can be a result of the built-in internal bias field *E_int_* generated by excess electrons and charge defects like oxygen vacancies. These electrons trapped near the interface with the bottom electrode can form defect dipoles aligned during the formation of domain structure, and result in an internal bias field oriented towards the electrode.

Domain structure in Co-, Ni-, Zn-, Nb-, and W-modified multiferroic BiFeO_3_ represented stripe domains with different orientations [100]. The domains studied by TEM were stable under the action of the electron beam, and their size did not vary much with the composition [100]. Obtained diffraction pattern in periodical domains revealed by high-resolution TEM showed apparent splitting of the electron diffraction spots perpendicular to the (110) planes, indicating the formation of the (110) domain walls [100].

### 4.4. BiFeO_3-x_LaFeO_3_-0.05La_2/3_TiO_3_

Domain structure in BiFeO_3-x_LaFeO_3_-0.05La_2/3_TiO_3_ ceramics has been separated into domains with different length scales [101]: fine scale ferroelectric/ferroelastic twin domains (10–20 nm) and larger regions (100–200 nm), which define the domain structure associated with antiphase tilting. This fact suggests that the local direction of polarization and strain are inconsistent with the rhombohedral distortion of the macroscopic tilt system and symmetry. According to the proposed model, each tilt domain consisted of many tens of finer scale ferroelastic/ferroelectric twins (10–20 nm), which average polarization vector and spontaneous strain are consistent with the symmetry of the macroscopic tilt system, but with lower local symmetry.

### 4.5. Temperature Dependence of Local Piezoelectric Response

As expected, the values of piezoelectric coefficients in RE-doped BFO ceramics decrease significantly with decreasing temperature [69,102]. Stabilization of the ceramics in a wide temperature range and improvement of the properties at close to room temperature is very important for various applications [103]. Pure BFO ceramics demonstrate a significant increase of the piezoelectric and dielectric properties at elevated temperatures [104]. The value of piezoelectric charge coefficient d_33_ measured at 1 Hz ranged from 33 pm/V at 24 °C to 118 pm/V at 262 °C. Dielectric permittivity demonstrates a similar trend and reaches a value above 3000 at elevated temperatures and low frequencies. This effect was attributed to Maxwell–Wagner relaxation from the grain boundaries and reversible motion of the conductive non-180° domain walls. The addition 9% of Dy stabilized piezoelectric properties up to 350 °C, while BFO doped with Sm and Gd demonstrated strong decrease of d_33_ at temperatures above 200 °C [69].

### 4.6. Grain Size–Domain Size Relation

Generally, the properties of ferroelectric and piezoelectric ceramics strongly depend on the grain size [105]. The effect of grain size on domain structure, dielectric, and piezoelectric properties in different materials has been extensively studied by Arlt and coworkers [60,106,107,108]. Castillo et al. [109] revealed that a decrease of the grain size in pure BFO ceramics led to an increase in the elementary cell volume and a decrease of the local piezoresponse. Grains with size below a few hundred nanometers are predominantly single domain [109]. At the same time, complex domain patterns including both 180° and ferroelastic domain walls are typical for micron-sized grains, similar to BaTiO_3_-based ceramics [60].

### 4.7. Local Switching by PFM

The main disadvantage of BFO ceramics for piezoelectric applications is the combination of high electrical conductivity and high coercive field [11]. Due to the high leakage current and low breakdown field, it is difficult to reach saturation during the poling process. Several authors reported non-saturated polarization-electric-field (P-E) loops, which are often misinterpreted because of the high contribution of the conductive current [11]. Thus, the local measurement of piezoresponse hysteresis and local switching by the SPM tip look like an attractive method for studying polarization reversal properties in BFO ceramics [21,97].

**Figure 8:**
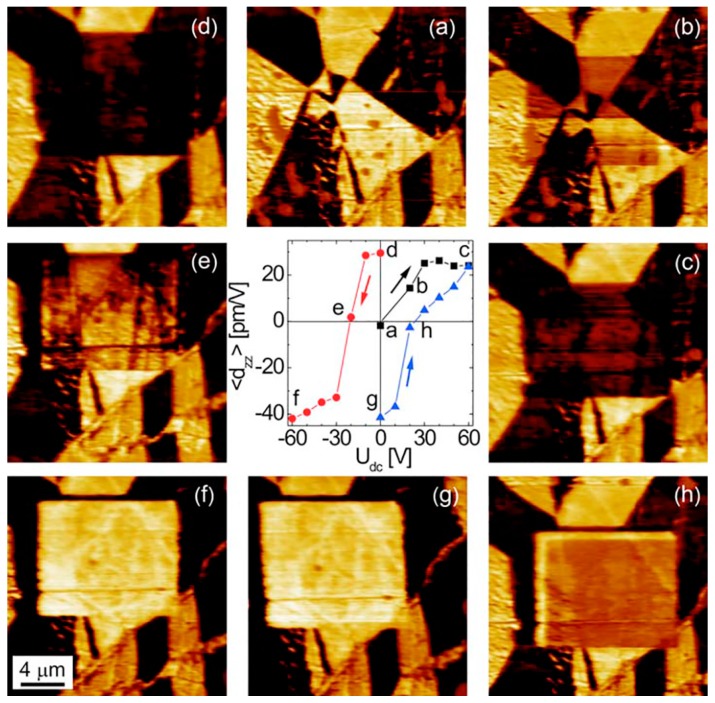
Local switching by progressive step-by-step poling of the area. Out-of-plane PFM images of BiFeO_3_ ceramic after poling an inner area of 10 × 10 μm^2^ subsequently at different bias voltages: (**a**) Virgin state U_dc_ = 0; (**b**) +20; (**c**) +60; (**d**) 0; (**e**) −20; (**f**) −60; (**g**) 0; and (**h**) +20 V. The average PFM signal of this area after these and more poling procedures in zero bias yields a hysteresis curve vs. U_dc_ (central inset). Adapted from [89], with permission from © 2007 AIP Publishing LLC.

The original approach was proposed in BFO for the study of polarization reversal during successive poling of a square area (10 × 10 μm^2^) under stepwise-increasing DC voltage (Figure 8) [89]. After each poling procedure, the 20 × 20 μm^2^ area was scanned again without DC bias. Thus, an analog of the macroscopic polarization reversal was realized, and the main characteristic of this poling process could be extracted from the average piezoelectric signal inside the poling square versus applied DC bias.

Another approach for the evaluation of the switching properties of doped BFO-based ceramics was demonstrated in Reference [93]. The bi-domain structure was created in a single grain using ±30 V DC voltage bias voltage and scanned by PFM. After that, the piezoresponse histograms of poled BFO and co-substituted samples were created to evaluate the average piezoresponse value. The dependence of the difference between negative and positive piezoelectric signals on the concentration of Sc dopant demonstrated a maximum for 1% Sc and minimum for the 7% Sc co-substituted sample.

## 5. Domain Structure and Local Piezoelectric Properties of KNN-Based Ceramics

### 5.1. Domain Structure before Poling

**Figure 9:**
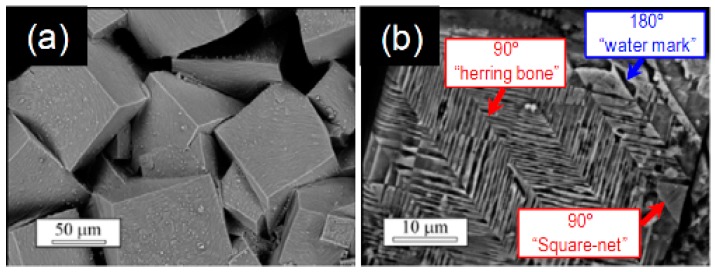
Scanning electron microscopy of the (**a**) grain structure and (**b**) domain structure revealed by chemical etching in KNN ceramics. Adapted from [59], with permission from © 2012 Royal Society of Chemistry.

**Figure 10:**
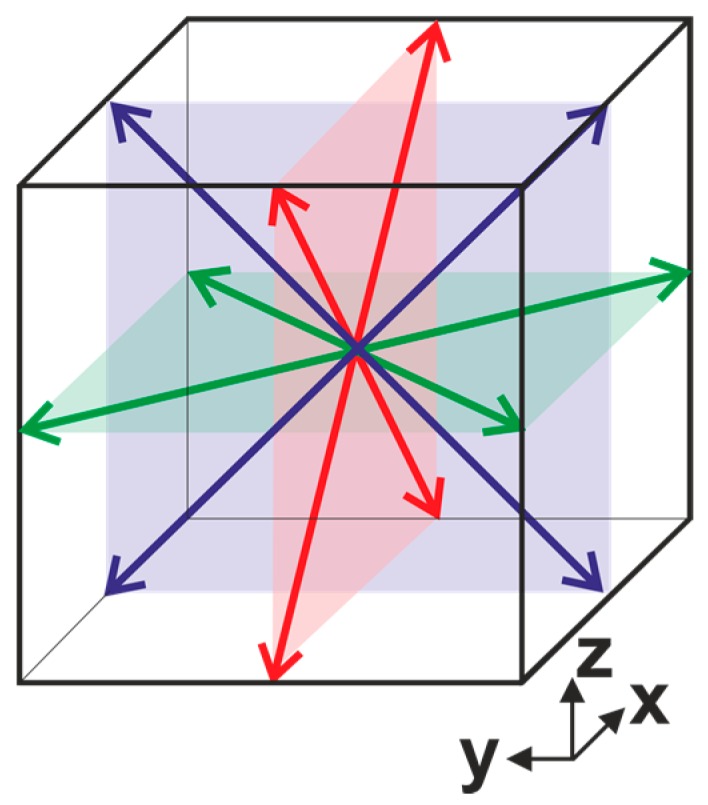
The schematic diagram of KNN crystal structure and possible directions of the spontaneous polarization.

The typical grain structure of KNN ceramics is represented by cubic grains with faceted grain boundaries (Figure 9a) [110]. Such ceramics possess orthorhombic symmetry at room temperature, which suggests the existence of 60°, 90°, 120°, and 180° domain walls in the *Bmm2* structure [67,111]. The spontaneous polarization Ps of the orthorhombic phase is parallel to the {100}_C_ direction, and 12 polarization orientations are permissible in the orthorhombic phase (Figure 10). Using pseudocubic coordinates, charged and uncharged 90° domain walls are confined to {100}_C_ planes, while the charged 60° domain walls and uncharged 120° domain walls are limited to {110}_C_ planes [67]. The 180° domain walls are oriented parallel to the spontaneous polarization Ps. The indices of uncharged 60° domain walls and charged 120° domain walls depend on the piezoelectric or electrostrictive coefficients [112,113]. Using pseudocubic coordinates, charged and uncharged 90° domain walls are confined to {100}_C_ planes, while the charged 60° domain walls and uncharged 120° domain walls are limited to {110}_C_ planes [67]. The 180° domain walls are oriented parallel to the spontaneous polarization *P_s_*. The indices of uncharged 60° domain walls and charged 120° domain walls depend on the piezoelectric or electrostrictive coefficients [112,113].

The most usual domain structure types in KNN ceramics are so called “watermarks” and “herringbone” [60,67,70] (Figure 9b). However, the existence of “zigzag” and “square net” are also reported in a few publications [59,114]. Such structures result from free energy minimization. The herringbone pattern contains parallel strips subdivided by narrow V-shaped domains, which form a 120° angle bisected by the long domain wall. The walls for both short lamellar domains and long domains are oriented along {211} directions [70]. Domain structure was found to be dependent on the average grain size [115]. According to the thermodynamic theory [116], the equilibrium domain size is proportional to the square root of domain wall energy, since domain size is determined by a balance between the energy of domain wall and the energies of depolarization and elastic fields caused by the spontaneous polarization and strain [117]. It was shown in KNN that the fine grains exhibit predominant lamellar twinning, while in coarse grains, twinning with a banded structure released [118]. PFM study of KNN-based ceramics also revealed a non-zero piezoresponse in the unpoled state (self-polarization effect) [119].

### 5.2. Domain Structure after Poling

The domain structure in most grains becomes periodical after poling (Figure 3a–f). Some grains exhibit only a simple parallel set of stripes extending over the whole grain, while other grains exhibit two or more sets of parallel domain stripes [67,120]. This domain structure was associated with the formation of ferroelastic orthorhombic domains, termed 60°, 90°, or 120° domains [67]. The average domain width is strongly dependent on the grain size, ranging from 100 nm to 1–3 μm [67]. When several parallel domain stripes exist in one polycrystalline grain, the intersection angles formed between the adjacent sets of parallel domain stripes are either around 45° or 135°. According to the intersection angles, these domain patterns were classified as domain configuration of type I and type II, respectively (Figure 3g,h) [67]. Two models were proposed to explain such domain patterns. One is composed of 90°, 60°, and 120° domain walls; the other one is composed of 180°, 90°, and 120° domain walls. However, diverse crystallographic planes can be observed at the polishing plane due to the random orientation alignment of the polycrystalline grains, and values of the intersection angle between sets of parallel domain stripes may also show a large variation [67].

**Figure 11:**
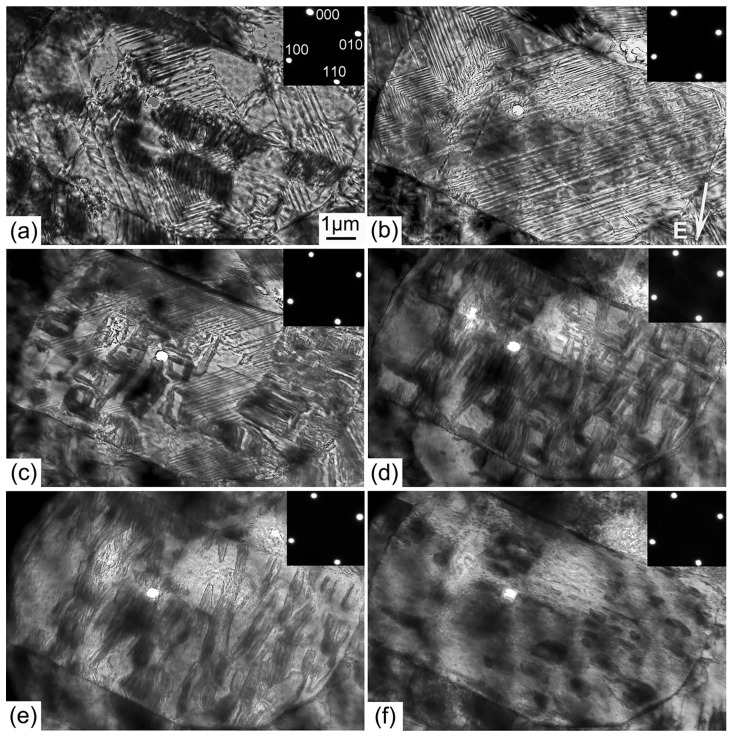
In situ TEM observation during electrical poling: (**a**) 8 kV/cm; (**b**) 10 kV/cm; (**c**) 14 kV/cm; (**d**) 18 kV/cm. The direction of poling fields is indicated by the bright arrow in (**b**). Representative selected area diffraction pattern at each poling field are shown in the insets. Adapted from [70], with permission from © 2013 AIP Publishing LLC.

The poor stability of piezoelectric properties (aging) found in (K_0.50_Na_0.50_)_0.935_Li_0.065_NbO_3_ and (K_0.50_Na_0.50_)_0.92_Li_0.08_NbO_3_ ceramics is due to the depoling effect attributed to the formation of 180° domains (Figure 11) [70]. The reorientation of 90° domains is much more difficult than that of the 180° domains in orthorhombic phase, being practically forbidden for 90° single domains due to clamping [70]. The formation of 180° domains is commonly attributed to the reduction of electrostatic energy, and their presence significantly simplifies the depoling process.

### 5.3. Coexistence of Tetragonal and Orthorombic Phases

**Figure 12:**
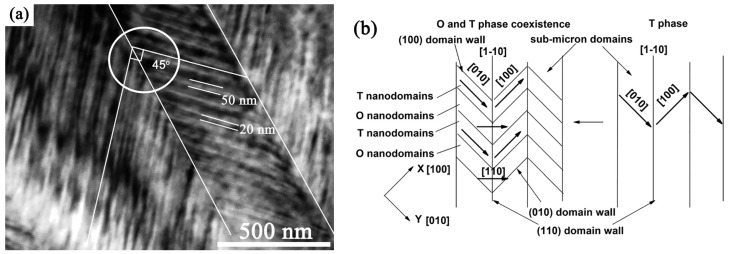
(**a**) Bright-field image of NKNS-0.0375LT ceramic sample; (**b**) Scheme of domain morphology evolution from single T phase to coexisted O and T phases. Adapted from [121], with permission from © 2011 AIP Publishing LLC.

The coexistence of orthorhombic and tetragonal phases in KNN ceramics was found to depend on the doping conditions and phase content [59,70,121]. Domain patterns in poled samples containing these two phases consist of lamellar domains that are slightly bent, and therefore, domain walls deviate from straight lines (Figure 12a). Such domains are usually discussed in terms of lattice distortion between the orthorhombic and tetragonal crystal phases [121].

**Figure 13:**
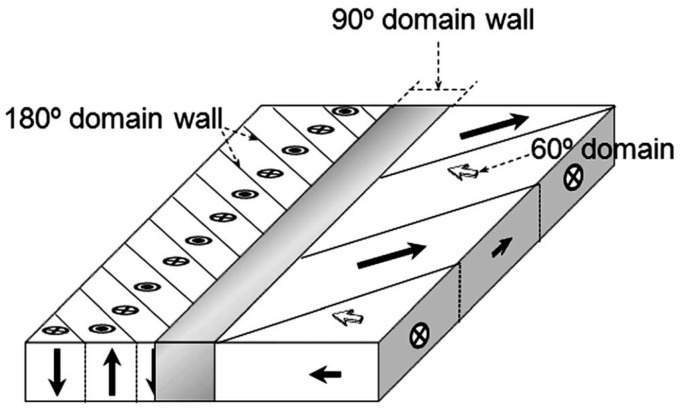
Schematic representation of the three-dimensional domain structure. Adapted from [59], with permission from © 2012 Royal Society of Chemistry.

The polymorphic phase boundary typically results in a decrease of polarization rather than in an enhancement of dielectric and piezoelectric properties, as for MPBs in piezoceramics [59]. One of the possible current understandings of the origin of the polymorphic phase boundary in KNN-type materials is based on specific domain distribution [59]. A schematic of domain structure formed at the PPB is represented in Figure 13 [59]. This shows two striped regions separated by a 90° domain wall. The striped region on the left-hand side contains 180° domains with the polarization direction perpendicular to the surface, whereas on the right-hand side, an alternation of tetragonal and orthorhombic phases is present, giving rise to 60° or 120° domains. The high-temperature tetragonal domain structure serves as a template that constrains the formation of the orthorhombic domains and eventually preserves the tetragonal structure by compensating the surface energy of the ceramic grains. In addition to the stability of the tetragonal symmetry at temperatures below that of the T_O–T_ transition, the unusual relaxor behavior of this transition was also explained. The proposed domain structure clearly explains a reduction of the piezoelectric properties in lead-free ceramics due to the limitation of the domain dynamics. On the other hand, excellent properties of the composition proposed by Saito et al. [31] were attributed to the following facts: (1) T_O–T_ transition is shifted close to room temperature, thus the ceramic samples have high tetragonality ratio; (2) texturing of the ceramics results in an excellent path for stress relief by the alignment of polar orientations and probably by increasing the grain size that contributes to better accommodation of stresses.

### 5.4. Temperaute Dependences of Local Piezoelectric Response and Grain Size–Domain Size Relation

**Figure 14:**
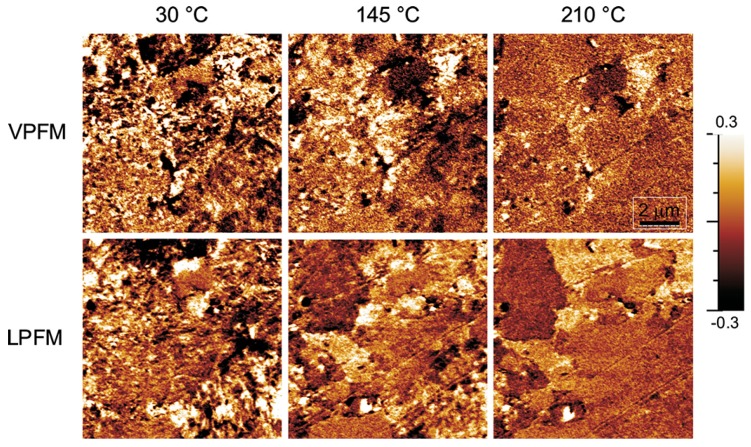
Vertical and lateral piezoresponse force microscopy (VPFM and LPFM, respectively) images of the polished surface of 0.95(Na_0.49_ K_0.49_Li_0.02_)(Nb_0.8_Ta_0.2_)O_3_-0.05CaZrO_3_ ceramics taken upon heating at 30 °C, 145 °C, and 210 °C. Adapted from [122], with permission from © 2014 AIP Publishing LLC.

Domain structure transformation as a function of temperature has been studied by PFM [122]. The transition into the tetragonal phase occurs at approximately 65 °C, and is followed up by the reconstruction of the domain structure in the temperature range between 50 and 130 °C. At higher temperatures, domains start to disappear first in larger grains, being more stable in smaller grains. Finally, at 210 °C, only some residual nanodomains are observed at the grain boundaries (Figure 14).

Domain structure transformation with temperature was also studied in 0.96(K_0.4_Na_0.6_)(Nb_0.96_Sb_0.04_)O_3_-0.04Bi_0.5_K_0.5_Zr_0.85_Sn_0.15_O_3_ ceramics [123]. The process leads to the reorganization of domain structures between 28 °C and 50 °C, resulting in different temperature-dependent behaviors of regular and irregular domains [123]. At high temperature (above 100 °C), the amplitude of piezoresponse significantly decreased, accompanied with obscured ferroelectric domain boundaries. At lower temperatures (50–100 °C), indiscriminate behaviors of the multi-scale domains may be related to the single-phase state (T state). The nano-scale domains possess improved flexibility due to the reduced domain wall energy, and could easily respond to external stimuli, contributing to the piezoelectric performance.

Li_0.02_(K_0.45_Na_0.55_)_0.98_NbO_3_ (LKNN) ceramics demonstrated a clear dependence of domain structure on the grain size [124]. A decrease of the period of 90° lamellar domains on decreasing grain size was observed down to 3 μm. Below 3 μm, an opposite dependence of domain period on grain size was found. The grain size effect was studied in 0.95(K_0.5_Na_0.5_)NbO_3_-0.05BaTiO_3_ (KNN-BT) ceramics with different secondary milling times, leading to different grain sizes. Reduction of the grain size from 14 to 1 μm increased the maximum dielectric permittivity from 2600 to 5000. This fact was attributed to high internal non-uniformly distributed stress in grains resulting in an easier domain wall motion [115].

### 5.5. Local Switching by PFM

Dependence of the local domain switching on the domain structure period was observed in Reference [82]. It was demonstrated that the decrease of the average domain size was accompanied by the decrease of coercive voltage [115]. The temperature increase significantly modified hysteresis loop shape [122]. The remnant and maximal piezoresponse reached their peak values at approximately 55 °C, which was attributed to the orthorhombic–tetragonal transition that occurred in the studied composition at approximately 65 °C.

Rapid decrease of the piezoresponse amplitude and remarkable broadening of the local loops were obtained in the tetragonal phase. It was assumed that nucleation of new domains contributed to polarization reversal, meaning higher remnant polarization and maximal switchable piezoresponse. The transition into the tetragonal state was accompanied by a change in local polarization switching kinetics: the domain nucleation under the PFM tip occurred at a larger bias voltage, while propagation of the new domain was faster than in the orthorhombic phase. The less saturated local piezoelectric hysteresis loops and increased difference between the maximal and remnant piezoresponse indicated that the switching was incomplete and unstable. Thus, higher temperature promoted the effect of backswitching of the reversed domains.

## 6. Domain Structure Input to the Dielectric Permittivity and Piezoelectricity

Relation between domain structure and dielectric permittivity in wide frequency range is well known in piezoelectric ceramics [107,125,126,127,128]. A number of models were suggested to describe the mechanism of the effect:

1. Domain wall vibrations under the action of applied electric field (resonance domain wall oscillations) [127,128,129,130] described by empirical Rayleigh law [126,127,128].
2. Emission of elastic shear waves from ferroelastic domain walls [129,131,132,133,134,135]. Ferroelastic domain walls are displaced in an applied electric field, which causes a shift of matter on both sides of the wall in opposite directions parallel to the domain wall. Thus, the domain walls behave like shear wave transducers; the shear waves emitted into the adjacent grains at high frequencies cause considerable dielectric losses, resulting in dielectric relaxation.
3. Piezoelectric sound generation by laminar stacks of 180° domains (piezoelectric resonance of domains) [133,134,135].
4. Maxwell–Wagner effect of the conductive domain walls [136].

However, some experimental data exists related to direct measurements of the influence of domain structure on the resulting properties of ferroelectric ceramics. Strong dielectric dispersion in the MHz to GHz range was found in pristine KNN ceramics, and was attributed to the dynamics of ferroelastic–ferroelectric domain walls [137]. The oscillation of sets of laminar ferroelastic domain walls was proposed to emit transverse acoustic waves [128]. The nonlinear dielectric response was found in KNN-0.05LT piezoelectric ceramics that underwent an abrupt fall at the polymorphic phase transition due to the change of domain configuration [137]. The dielectric Rayleigh coefficient in the tetragonal phase zone was smaller than that in the orthorhombic one. Additionally, during the polymorphic phase transition (PPT), both the intrinsic and the extrinsic contributions to the dielectric constant significantly fluctuated with temperature variation.

The nonlinear dielectric response at low frequencies and irreversible extrinsic contribution to dielectric permittivity found in KNN-LTS ceramics were strongly dependent on the domain configuration [130], determined by the crystallographic structure. The spontaneous distortion in tetragonal phase is larger than that in the orthorhombic one, which constrains the domain wall motion and results in smaller irreversible extrinsic contribution for the tetragonal compositions. The behavior in the PPT region is controlled by two effects: on one hand, the high domain wall concentration decreases the wall mobility due to self-clamping, while on the other hand, the additional spontaneous polarization emerging from the phase coexistence enhances the domain wall mobility. Due to the combination of both effects, the irreversible extrinsic contribution in the PPT is similar to that in the tetragonal region, and lower than that in the orthorhombic one. Dielectric permittivity demonstrated non-monotonic dependence on the domain wall concentration: first increasing with density, and then falling down [130]. The intrinsic dielectric permittivity increased, while irreversible extrinsic contribution decreased after poling [130].

In the Sb^5+^-modified KNN-based ceramics, domain-wall vibrations were found to change from the resonance to relaxation mode due to a substantial increase of damping constant [129]. Doping by Mn influences the mobility of ferroelastic domains [138]. Sufficiently strong domain motion was proposed due to clamping along the vertical direction to grain bars. Internal friction occurred near the grain boundaries and ferroelectric domain walls during polarization reversal in the external electric field. Therefore, the friction force significantly affects the dielectric response [139].

The role of domain structure and nanodomains appeared close to the PPT region in KNN ceramics remains poorly studied. Significant variation in the electrical and mechanical properties was observed in KNN before and after poling [140]. The highest coercive field and the lowest field-induced strain were obtained in the pristine samples of the PPB composition, while the opposite trend was detected after poling [140]. Unusual behavior of KNN ceramics close to PPT is usually attributed to the appearance of nanodomains due to two coexisting phases. High domain wall concentration leads to a larger domain wall energy barrier in the PPT region, making the domain wall motion difficult. Therefore, the coercive field of the pristine samples reaches a maximum in the vicinity of the PPT region [140]. During poling, the nanodomains transform irreversibly into micron-sized domains. The domains in the PPT region move more easily due to the existence of additional polarization states. Therefore, the coercive field of the poled ceramics has a minimum value at the PPT composition [140]. At the same time, it was proposed that nanodomains could be responsible for enhanced electric field-induced strain [141].

Hayati et al. found that the addition of nanosized ZnO additive increased the grain size of the KNN ceramics, with a corresponding increase of the domain size. This increase was accompanied with a reduction of the coercive field [142]. For higher doping levels, Zn^2+^ ions entered in B-positions, resulting in the formation of oxygen vacancies that pinned domain walls and concomitant increase of the coercive field [142].

**Figure 15:**
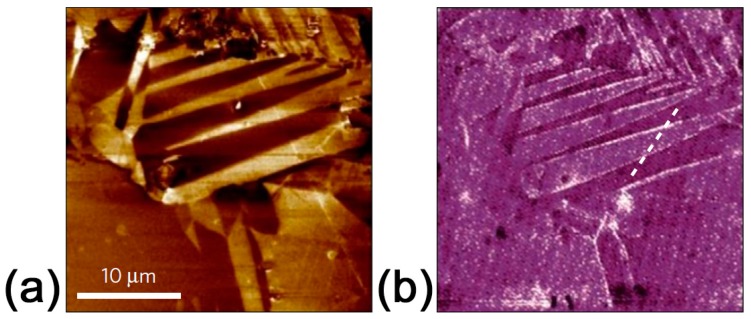
(**a**) Out-of-plane PFM amplitude and (**b**) conductive atomic force microscopy (c-AFM) images of selected regions with domains in BiFeO_3_ annealed in O_2_ atmosphere. Adapted from [7], with permission from © 2016 Nature Publishing Group.

An exhaustive discussion about indirect measurements of domain wall input to dielectric and piezoelectric properties of BFO was reviewed by Rojac and co-authors [11]. The poling of rhombohedral BiFeO_3_ ceramics through 71° and 109° domain wall reorientation was shown and quantified using analysis of X-ray diffraction band intensities [11]. The non-linear effects related to the motion of conductive domain walls in BFO bulk ceramics (non-linear Maxwell–Wagner effect) were recently demonstrated [105]. The domain wall and grain boundary conductivity have been measured by conductive atomic force microscopy (Figure 15), and mobility of domain walls under sub-switching electric fields has been determined in BFO ceramics [7,136]. The high resolution STEM imaging of the individual domain walls was done together with inspection of their conductivity depends on the sintering conditions [7]. The conductivity of domain walls in bulk BFO ceramics was suggested to be p-type, related to the polaron hopping mechanism between Fe^4+^ and Fe^3+^ sites [7].

## 7. Conclusions

The domain dynamics plays an important role in the physical properties of lead-free piezoelectric ceramics properties, and, as such, should be rigorously studied. The rapid development of modern microscopic techniques gives the opportunity not only to visualize domain structure, but also to quantitatively determine the orientation and values of spontaneous polarization and piezoelectric coefficient locally. The morphotropic and polymorphic phase boundaries existing both in BFO and KNN are principally coined to the transformation of the domain assemblages and phase structure. Ferroelectric nanodomains and inhomogeneously distributed mixed phases appear due to doping and peculiarities of the sintering process, and are extremely important for future applications. This review is one of the first attempts to describe the domain structure and local piezoelectric properties and their impact on the macroscopic performance of lead-free piezoelectric ceramics based on BFO and KNN.
